# Etude du syndrome métabolique et l’activité physique chez une population de la ville de Marrakech, au Maroc

**DOI:** 10.11604/pamj.2021.38.21.20219

**Published:** 2021-01-11

**Authors:** Zineb Hannoun, Khouloud Harraqui, Rachmat Attoumane Ben Ali, Kamar Tahiri, Omar Ben Smail, Fatine El Arabi, Abdellatif Bour

**Affiliations:** 1 Laboratoire des Essais Biologiques, Equipe de Transition Alimentaire et Nutritionnelle (ETAN), Faculté des Sciences, Université Ibn Tofail, BP 133, Kenitra 14000, Maroc

**Keywords:** Maladies cardiovasculaires, syndrome métabolique, anthropométrie, activité physique, Maroc, Cardiovascular diseases, metabolic syndrome, anthropometry, physical activity, Morocco

## Abstract

**Introduction:**

le but de cette étude était de faire une corrélation entre le syndrome métabolique et le niveau de l'activité physique chez une population de Marrakech, au Maroc.

**Méthodes:**

l'étude a été menée à l'Hôpital régional Ibn Zohr de Marrakech. L'indice de masse corporelle (IMC) a été calculé pour évaluer le degré d'obésité de chaque sujet. Pour déterminer le niveau d'activité physique, nous avons utilisé la version courte du questionnaire IPAQ (International Physical Activity Questionnaire); Les paramètres sanguins ont été mesurés par un automate de biochimie. Toutes les analyses statistiques ont été effectuées à l'aide du logiciel SPSS.

**Résultats:**

au total, 300 sujets ont participé à l'étude, dont 57,3% de femmes et 42,7% d'hommes avec un sex-ratio de 0,74. L'âge moyen de notre population était de 51,6 ± 13,42 ans. Soixante-dix-neuf des participants (26,3%) avaient un syndrome métabolique, avec une prédominance féminine: 60 femmes (34,9%) et 19 hommes (14,8%). Il existe une relation significative entre le niveau d'activité physique et la présence de syndrome métabolique (p = 0,002), entre le niveau d'activité physique et l'IMC et le tour de taille (p < 0,001) et (p = 0,003) respectivement.

**Conclusion:**

les résultats approuvent l´association significative entre l´obésité, le syndrome métabolique et le niveau de l´activité physique, ce qui nous inciterait à encourager l´application des règles hygiéno-diététiques, notamment l´activité physique, qui reste l´une des meilleures actions de prévention contre cette pathologie.

## Abstract (en)

**Introduction:**

the aim of this study was to correlate the metabolic syndrome with the level of the physical activity in a population from Marrakech, Morocco.

**Methods:**

the study was conducted at Ibn Zohr Regional Hospital in Marrakech. The body mass index (BMI) was calculated to assess the degree of obesity of each subject. To determine the level of physical activity, we used the short version of the IPAQ (International Physical Activity Questionnaire); Blood parameters were measured by a Biochemistry Automaton. All statistical analyzes were performed using SPSS software.

**Results:**

a total of 300 subjects participated in the study, which 57.3% were female and 42.7% were male with a sex ratio of 0.74. The average age of our population was 51.6± 13.42 years old. Seventy-nine of the participants (26.3%) had a metabolic syndrome, with a predominance of female: 60 women (34.9%) and 19 men (14.8%). There is a significant relationship between level of physical activity and the presence of metabolic syndrome (P = 0.002), between physical activity level and BMI and waist circumference (p < 0.001) and (p = 0.003) respectively.

**Conclusion:**

the result shows a significant association between obesity, metabolic syndrome and the level of the physical activity, which would encourage us to encourage the application of lifestyle rules, including physical activity, which remains one of the best preventive actions against this pathology.

## Introduction

À l´échelle mondiale, la charge des maladies non transmissibles a augmenté rapidement, ceci porte sur les deux principaux facteurs de risque : une mauvaise alimentation et le manque d´exercice physique [1]. Le syndrome métabolique est une entité pathologique qui se traduit par, la présence chez un même individu, de plusieurs anomalies métaboliques, à savoir, l´obésité centrale, une hypertriglycéridémie, une baisse du HDL cholestérol, une hypertension artérielle et une intolérance au glucose qui finalement l´expose, trois fois aux risques cardiovasculaires et neuf fois au risque du diabète de type 2 [2].

Une diminution de l'activité physique est susceptible d'en être un facteur étiologique important. En se référant à l´OMS, l´activité physique est définie comme tout mouvement corporel produit par les muscles squelettiques, responsable d´une augmentation de la dépense énergétique. La sédentarité en revanche, est considérée comme le quatrième facteur de risque de décès dans le monde (6%), on estime qu´elle est la principale cause d'environ 21 à 25% des cancers du sein et du colon, 27% des cas de diabète et environ 30% des cas de cardiopathie ischémique [3]. Vu la relation étroite qu´elle a avec les facteurs de risque des maladies non transmissibles, pratiquer une activité physique régulière et adaptée s´est avéré donc, comme un paramètre essentiel pour le maintien d´un état de santé favorable [4].

L'objectif de cette étude était de faire une corrélation entre le syndrome métabolique et le niveau de l´activité physique chez une population de Marrakech, au Maroc.

## Méthodes

Il s’agit d’une étude épidémiologique réalisée sur une population de la ville de Marrakech au Sud-ouest du Maroc, s´allongeant du mois de mars au mois de juillet 2018 sur, au total 300 sujets dont 42,7% des hommes et 57,3% des femmes, âgés de plus de 18 ans. La première étape était l´obtention d´une autorisation du Ministère de la Santé signée par le délégué régional du Ministère de la Santé, le directeur de l´hôpital et le chef du laboratoire pour mener l'étude à l'hôpital Ibn Zohr de Marrakech. Les sujets recrutés étaient des individus qui venaient à l´hôpital simplement pour un contrôle ou pour un bilan médical général.

Avec l´aide des infirmiers, nous leur avons expliqué le but de l'étude et puis ils se sont portés volontaires et ont signé le formulaire de consentement. Puis ils ont rempli individuellement le questionnaire IPAQ et les informations sociodémographiques (âge, sexe, niveau d'instruction, état matrimonial et emploi). Nous avons pris la responsabilité d'enregistrer les réponses des sujets analphabètes nous-mêmes, selon leurs propres réponses. Les mesures anthropométriques ont été prises dans une salle réservée pour eux. Leur confidentialité et leur vie privée ont été respectées. L´éthique est allouée à la délégation régionale du Ministère de la Santé qui garantit à ces enquêtés comme bénéfice de les orienter et de les prendre en charge en fonction de leur(s) pathologie(s); si pathologie il y a. Ce qui rendra notre étude éthiquement acceptable.

**Critères d´inclusion:** les personnes dont l´âge est de 18 ans et plus; Les deux genres; personnes résidentes exclusivement à Marrakech.

**Critères d´exclusion:** femmes enceintes; femmes allaitantes; personnes atteintes de pathologies de la thyroïde; les non résidants à la ville de Marrakech.

Le poids en kilogramme est mesuré à l´aide d´une balance mécanique de marque SECA, avec une précision de 0,5 Kg. La mesure de la taille est effectuée à l´aide d´une micro toise murale de marque STANILEY. L´indice de masse corporel (IMC) a été calculé pour évaluer le degré d´obésité de chaque sujet. Le tour de taille a été mesuré à l´aide d´un mètre ruban au nombril. La pression artérielle a été enregistrée en position assise après 15 minutes de repos, à deux intervalles de 5 minutes; ces mesures ont été effectuées à l´aide d´un sphygmomanomètre à mercure standard sur le bras droit et la moyenne des deux mesures a été enregistrée pour la comparaison. Pour déterminer le niveau d'activité physique, nous avons utilisé la version courte du questionnaire IPAQ (International Physical Activity Questionnaire).

Pour chaque sujet à jeun (12 à 14h de jeûne), nous avons prélevé un flacon du sang veineux. Les prélèvements sanguins se faisaient de 8: 00 à 10: 30 le matin, et ils étaient assurés par les infirmiers de l´hôpital. Ensuite les paramètres sanguins ont été mesurés par un automate de biochimie adéquat après 10 min de centrifugation. Les analyses biologiques ont été effectuées par les techniciens du laboratoire sous la supervision du directeur régional de la région de Marrakech. Le diagnostic du syndrome métabolique a été choisi selon la définition du National Cholesterol Education Program Adult Treatment Panel III (NCEP-ATP III) et qui nécessite l'association d'au moins trois des cinq critères suivants: tour de taille chez les femmes ≥ 88 cm et chez les hommes ≥ 102 cm, hypertriglycéridémie ≥ 1,5 g/L, HDL cholestérol < 0,50 g/L chez les femmes et < 0,40 g / L chez les hommes, pression artérielle ≥ 130/85 mmHg, glycémie à jeun ≥ 1,1 g/L (ou être sous traitement médicamenteux des anomalies précitées) [5]. Les examens biologiques et cliniques de ces personnes ont été récupérés à partir des registres des analyses et des mesures cliniques de l´hôpital Ibn Zohr. Ces analyses appartiennent à ces personnes, objets de notre enquête.

Pour effectuer des analyses statistiques, nous avons utilisé SPSS (Statistical Package for Social Sciences) version 23.0. Les variables quantitatives ont été décrites en utilisant la moyenne et l'écart type ; tandis que les variables catégorielles ont été décrites en utilisant les nombres et les pourcentages. Nous avons utilisé le test du Chi-deux X^2^ pour évaluer l'association entre deux variables catégorielles. Le test est considéré comme significatif lorsque la valeur de p est inférieure à 0,05.

## Résultats

### Activité physique et paramètres anthropométriques

Le Tableau 1 montre qu´il existe une relation très significative entre le niveau d´activité physique et le tour de taille (P = 0,003) et entre le niveau d´activité physique et l´IMC (P < 0,001); Une plus grande majorité des sujets ayant un tour de taille élevé et qui sont obèses ont un niveau d´activité physique assez faible.

**Tableau 1:** résultats de l´association des différents niveaux d´activité physique et des paramètres anthropométriques (en pourcentage %)

	Intense	Modérée	Faible	Test Khi-deux
**Tour de Taille**				
Normal	33,3	24,4	12,3	P = 0,003
Elevé	66,6	75,5	87,7	
**IMC**				P <0,001
Dénutrition	0,0	0,0	1,5	
Maigreur	5,9	3,4	0,8	
Corpulence Normale	43,1	21,0	21,5	
Surpoids	41,2	48,7	33,1	
Obésité	9,8	26,9	43,1	

IMC: Indice de Masse Corporel

### Activité physique et syndrome métabolique

Selon la définition du NCEP-ATP III, soixante-dix-neuf des participants (26,3%) avaient un syndrome métabolique; Le Tableau 2 montre qu´il existe une relation significative (P = 0,002) entre le niveau d´activité physique et la présence ou l´absence du syndrome métabolique. Quatre individus atteints de syndrome métabolique avaient une activité physique intense, 32 étaient modérément actifs et 43 étaient sédentaires.

**Tableau 2:** résultats de l´association des deux groupes, avec et sans syndrome métabolique et le niveau d'activité physique

Niveau d´AP	SM + (n = 79)	SM - (n = 221)	Test Khi-deux
Intense	4	47	P = 0,002
Modéré	32	87	
Faible	43	87	

SM +: Avec le Syndrome Métabolique; SM -: Sans le Syndrome Métabolique; AP: Activité Physique

### Activité physique et critères du syndrome métabolique

A l´exception des Triglycérides (P = 0,68), on constate qu´il existe une relation significative entre quasiment tous les autres critères de définition du syndrome métabolique et le niveau d´activité physique. Les données sont décrites dans le Tableau 3.

**Tableau 3:** résultats de l´association des différents niveaux de l´activité physique et les critères du syndrome métabolique

		Niveau d´activité physique			
Critères SM		Intense	Modéré	Faible	Test Khi-2
**HTA**	Oui	10	48	67	P < 0,001
	Non	41	71	63	
**HG**	Oui	20	72	83	P = 0,009
	Non	31	47	47	
**HTG**	Oui	12	33	39	P = 0,68
	Non	39	86	91	
**HDL-C bas**	Oui	8	44	51	P = 0,008
	Non	43	75	79	
**TT Elevé**	Oui	34	90	114	P = 0,003
	Non	17	29	16	

AP: Activité Physique; SM : Syndrome Métabolique ; HTA : Hypertension Artérielle; HG: Hyperglycémie; HTG: Hypertriglycéridémie; ↙HDL-C: HDL-Cholestérol Bas; TT↗: Tour de Taille Elevé

## Discussion

### Analyse et discussion de la présence du syndrome métabolique

La fréquence du syndrome métabolique au sein de notre population, définie selon les critères du NCEP-ATP III était de 26,3% avec une prédominance féminine: 60 femmes (34,9%) et 19 hommes (14,8%); Cette différence en fonction du sexe était statistiquement très significative (P < 0,001). Des résultats comparables ont été rapportés: 21,6% en Sfax - Tunisie [6], 20,0% à Oran - Algérie [7] et 14,9% à Ottawa - Canada [8].

### Analyse et discussion de l’association entre le niveau d´activité physique, le syndrome métabolique et ses critères

Dans notre étude, la corrélation entre le tour de taille, l´IMC et le niveau d´activité physique est statistiquement très significative, (P = 0,003) et (P < 0,001) respectivement. L'étude de Shamra [9] concorde également avec nos résultats où le niveau d'AP est fortement lié à l'IMC. L'étude a révélé que les sujets sédentaires atteints de SM avaient un tour de taille élevé par rapport à une activité physique modérée. Selon la classification de l'IMC, l'obésité était plus fréquente chez les sujets sédentaires que chez les sujets présentant un syndrome métabolique et physiquement en activité modérée.

Une relation a été observée entre le niveau d´activité physique et le nombre de personnes ayant un syndrome métabolique. En effet, 43 personnes (p = 0,002) ayant un syndrome métabolique avaient une activité physique faible, ces résultats concordent avec ceux de Laraqui *et al*. [10] où 86,3 % des personnes ayant un syndrome métabolique n´avaient pas une activité physique régulière. Samara [11] dans son étude de la prévalence du syndrome métabolique par rapport à l´activité physique à la ville de Salé (Maroc) a fait les mêmes conclusions. Notre étude s´intègre dans le projet Obe-Maghreb, où une autre étude [12] a montré que les femmes des villes de Rabat-Salé pratiquent peu d´activités d´intensité modérée ou forte.

A l´exception des Triglycérides (p = 0,68), on constate qu´il existe une relation significative entre quasiment tous les autres critères du syndrome métabolique et le niveau d´activité physique. Chaque élément du syndrome métabolique peut être amélioré par l´activité physique régulière: dans les études d´intervention, l´activité physique diminue la graisse viscérale [13], augmente le HDL cholestérol et diminue les triglycérides [14], diminue la pression artérielle [15] et augmente l´insulinosensibilité [16-19]. Un nombre croissant des études récentes [20, 21] soutiennent l´idée que l´activité physique pourrait à elle seule améliorer, de manière directe et/ou indirecte, la sensibilité à l´insuline et atténuer voire supprimer le syndrome métabolique ou ses composantes via des mécanismes indépendants ou synergiques. De plus, cette amélioration de la sensibilité à l´insuline pourrait se faire indépendamment de la perte de poids [22-24].

Cependant, différentes formes d´activité physique peuvent influencer directement la résistance à l´insuline en provoquant, entre autres, une activation à court terme des transporteurs de glucose (GLUT-4) et de leurs récepteurs [25], en abaissant le taux circulant d´insuline [26, 27] et en augmentant la production des facteurs anti-inflammatoires tels que l´adiponectine [28, 29] ou encore en induisant des modifications myofibrillaires appropriées (dont une augmentation de la quantité de fibres oxydatives et insulino-sensibles) [30]. La cible principale des programmes d´activité physique devrait être l´insulino-résistance (qui est un paramètre prépondérant du syndrome métabolique), même si une activité physique régulière peut directement agir sur les autres facteurs que sont la pression artérielle [31, 32].

## Conclusion

Dans notre étude, les résultats approuvent l´association de manière très significative entre l´obésité, le syndrome métabolique et le niveau d´activité physique, ce qui nous inciterait à encourager l´application des règles hygiéno-diététiques, notamment l´activité physique, qui reste l´une des meilleures actions de prévention contre cette pathologie.

### Etat des connaissances sur le sujet

- Le syndrome métabolique est un ensemble de troubles métaboliques et de facteurs de risque de maladies cardiovasculaires et de résistance à l´insuline;
- Le manque d´exercice physique augmente la charge des maladies non transmissibles.

### Contribution de notre étude à la connaissance

- Nous avons connu la relation entre l´obésité, le syndrome métabolique et le niveau de l´activité physique d´une population de la ville de Marrakech ;
- Nos résultats nous informent sur l'urgence de l´application d'une stratégie d´un mode de vie sain, notamment en activité physique, pour arrêter la progression du syndrome métabolique et ses complications.

## Remerciements

Mes remerciements vont à tous ceux qui ont contribué directement ou indirectement à la réalisation de ce travail. Mes chaleureux remerciements vont aussi au Ministère marocain de la Santé et à l´ensemble de l´équipe du laboratoire d´analyses médicales du Centre Hospitalier Régional de Marrakech qui ont fait que ce travail se déroule dans les meilleures conditions.
